# Apigenin Loaded Lipoid–PLGA–TPGS Nanoparticles for Colon Cancer Therapy: Characterization, Sustained Release, Cytotoxicity, and Apoptosis Pathways

**DOI:** 10.3390/polym14173577

**Published:** 2022-08-30

**Authors:** Mohamed A. Alfaleh, Anwar M. Hashem, Turki S. Abujamel, Nabil A. Alhakamy, Mohd Abul Kalam, Yassine Riadi, Shadab Md

**Affiliations:** 1 Department of Pharmaceutics, Faculty of Pharmacy, King Abdulaziz University, Jeddah 21589, Saudi Arabia; 2 Vaccines and Immunotherapy Unit, King Fahd Medical Research Center, King Abdulaziz University, Jeddah 21589, Saudi Arabia; 3 Department of Medical Microbiology and Parasitology, Faculty of Medicine, King Abdulaziz University, Jeddah 21589, Saudi Arabia; 4 Department of Medical Laboratory Sciences, Faculty of Applied Medical Sciences, King Abdulaziz University, Jeddah 21589, Saudi Arabia; 5 Nanobiotechnology Unit, Department of Pharmaceutics, College of Pharmacy, King Saud University, Riyadh 11451, Saudi Arabia; 6 Department of Pharmaceutical Chemistry, College of Pharmacy, Prince Sattam Bin Abdulaziz University, Al-Kharj 11942, Saudi Arabia

**Keywords:** hybrid nanoparticle, apigenin, sustained release, mTOR, apoptosis, colon cancer

## Abstract

Colon cancer (CC) is one of major causes of mortality and affects the socio-economic status world-wide. Therefore, developing a novel and efficient delivery system is needed for CC management. Thus, in the present study, lipid polymer hybrid nanoparticles of apigenin (LPHyNPs) was prepared and characterized on various parameters such as particle size (234.80 ± 12.28 nm), PDI (0.11 ± 0.04), zeta potential (−5.15 ± 0.70 mV), EE (55.18 ± 3.61%), etc. Additionally, the DSC, XRD, and FT-IR analysis determined drug entrapment and affinity with the selected excipient, demonstrating a promising drug affinity with the lipid polymer. Morphological analysis via SEM and TEM exhibited spherical NPs with a dark color core, which indicated drug entrapment inside the core. In vitro release study showed significant (*p* < 0.05) sustained release of AGN from LPHyNPs than AGN suspension. Further, the therapeutic efficacy in terms of apoptosis and cell cycle arrest of developed LPHyNPs against CC was estimated by performing flow cytometry and comparing its effectiveness with blank LPHyNPs and AGN suspension, which exhibited remarkable outcomes in favor of LPHyNPs. Moreover, the mechanism behind the anticancer attribute was further explored by estimating gene expression of various signaling molecules such as Bcl-2, BAX, NF-κB, and mTOR that were involved in carcinogenic pathways, which indicated significant (*p* < 0.05) results for LPHyNPs. Moreover, to strengthen the anticancer potential of LPHyNPs against chemoresistance, the expression of JNK and MDR-1 genes was estimated. Outcomes showed that their expression level reduced appreciably when compared to blank LPHyNPs and AGN suspension. Hence, it can be concluded that developed LPHyNPs could be an efficient therapeutic system for managing CC.

## 1. Introduction

Colon cancer (CC) is among the leading causes of death worldwide, including lung cancer, breast cancer, and pancreatic cancer. In terms of diagnosis, it ranks 3rd (9.1%), whereas, in terms of the rate of mortality, it ranks 2nd (9.2%). As per the published report of 2020, approximately 1.9 million cases of CC were reported, and further global burden from CC may increase by 60%, i.e., ~2.2 million, by the end of 2035 [1]. Published reports have highlighted the fact that the level of incidence of CC is strongly correlated with the country’s socio-economic development as well as the lifestyle of the population [2]. Coexisting metabolic syndrome, obesity, red meat consumption, alcohol, and sedentary lifestyle are some of the driving forces for the increased cases of CC. It was found that the majority of CC cases are associated with mutation-related polyposis MUTYH (MAP), Lynch syndrome (HNPCC), familial adenomatous polyposis (FAP), and hamartomatous polyposis syndromes. The mean age of diagnosis of disease ranges between 44 and 61 year and inflammatory bowel disease, Crohn’s disease, as well as ulcerative colitis increase the risk of CC [3].

Cellular and molecular investigation showed the diverse role of multiple signaling pathways in its etiology. Among all, NF-κB/PI3K/Akt/mTOR/p53/BAX/JNK pathways have played a pivotal role [4]. NF-κB is one of the extensively explored proinflammatory transcription factors, but studies have shown its critical involvement in the etiology of colon and other types of cancer via increased proliferation, angiogenesis, metastasis, and reduced apoptosis [5]. In normal physiological conditions, NF-κB is present/restricted in the cytoplasm under the influence of the IKK complex via the IκB proteins. However, under the oncogenic stimulus, IKK gets activated, and IκB gets phosphorylated and degrades, leading to its nuclear translocation and subsequent initiation of the pro-oncogenic activity. Specifically, increased activity of Akt/PI3K pathways has been reported in CC. Akt is a critical regulator of tumor cell proliferation and survival and causes phosphorylation of various pro-oncogenic targets.

On the one hand, Akt causes nuclear translocation of NF-κB via phosphorylation of IKKα, while, on the other hand, it causes phosphorylation and activation of mTOR (mammalian target of rapamycin) [6]. Not only this, the direct relation between IKKα and mTOR were reported by Dan et al. 2007, where IKKα remains in association with TORC1 and controls the kinase activity of mTOR in tumorigenesis [7]. A significant role of NF-κB/Akt/mTOR pathways in tumorigenesis was confirmed by various preclinical studies where increased activity and level of PTEN (inhibitor of Akt) and rapamycin (inhibitor of mTOR) showed the anticancer effect [8]. Apart from the significant role of the NF-κB/mTOR pathway, reduced apoptosis via JNK/p53/BAX/Bcl-2 is also a decisive factor in the etiology of CC. In the CC and other types of cancer, reduced apoptosis is commonly reported, and any drug that stimulates or increases the apoptosis is considered a potential anticancer drug [9].

It is also important to note that various anticancer drugs were developed to manage and treat CC, but chemotherapeutic resistance is one of the leading factors for the poor clinical outcome of these drugs among the treated patients. One of the major reasons for the drug resistance in the CC is the increased expression of the multidrug resistance (MDR1) gene. MRD1 encodes the p-gp, also known as ATP-binding cassette subfamily B member 1 (ABCB1), and causes efflux of most anticancer drugs, leading to reduced bioavailability and drug resistance, and poor clinical outcomes [10]. Hence, in the present study, we designed and developed an apigenin (AGN) hybrid nanoparticle (HyNP) to manage and treat CC. 

AGN is also known as 4′,5,7-trihydroxyflavone and belongs to the class of compounds known as flavones. AGN is commonly found as an active constituent in many Chinese traditional systems of medicines. It possesses significant antioxidant, anti-inflammatory, and antitumor potential in various preclinical studies. The first report on the anticancer potential of AGN was reported by Bart et al., in 1986, and since then, numerous research has been done to explore its anticancer potential in various types of cancers [11]. AGN exhibits an anticancer effect via apoptosis induction where it modulates the level of caspases, BAX, Bcl2, p53 [12], etc. AGN also modulates cell cycle progression via blocking the cycle arrest at G2/M or G0/G1 checkpoint. Moreover, AGN induces autophagy, blocks migration and invasion, and inhibits angiogenesis. Considering the molecular anticancer mechanism of AGN, it modulates the PI3K/AKT/mTOR signaling pathway, NF-κB/MAPK/ERK pathway, and Wnt/JNK pathway [13].

Despite being a potent anticancer molecule, AGN suffers from significant pharmacokinetic limitations. As per Biopharmaceutical Classification System, AGN has been categorized as a Class II drug. It has high permeability but low solubility [14]. In the pharmacokinetic study, it was found that upon administration of 60 mg/kg of AGN in rats, C_max_ was 1.33 ± 0.24 μg/mL, and AUC_0–t°_ was found to be 11.76 ± 1.52 μg h/mL [15]. Thus, to over the limitation, AGN-loaded lipid-polymer-HyNP (LPHyNPs) was fabricated and explored for anticancer potential in the in vitro model of CC. 

It is further important to understand that lipid-based NPs, because of having an amphiphilic chain, easily get functionalized, become biocompatible, and possess increased duration of circulation, but at the same time suffers from the limitations of instability and loss of structural integrity [16]. So, polymeric NPs, which are one of the advanced drug delivery systems that enhanced stability and increased drug loading capacity was selected. Thus, attempts were made to combine both the drug delivery system and names as lipid–polymer HyNPs [17,18]. This drug delivery system possesses a polymeric core and lipidic shell where the lipid core lies on the outer side, retards the degradation of polymers via restraining inward diffusion of water. As per the report of Yu et al., 2018, LPHyNPs loaded with salinomycin showed improved pharmacokinetic attributes. Salzano et al., fabricated HyNPs and reported the controlled release of daunorubicin and lornoxicam [19,20]. Additionally, hybrid PLGA NPs was fabricated, in which a stabilizer was also added. One commonly used stabilizer is D-α-tocopherol polyethylene glycol 1000 succinate (vitamin E-TPGS). When PLGA as a polymer and TPGS as a stabilizer were used, p-gp efflux was reduced considerably, and improved pharmacological attributes could be achieved [21]. 

Thus, the present study was designed with novelty to provide promising therapeutic effects against CC. In this case, AGN-loaded lipid HyNPs of Vit-E-TPGS (LPHyNPs) was fabricated. Further, it was prepared and characterized on various parameters such as particle size, polydispersity index (PDI), zeta potential, and EE. Additionally, LPHyNPs was evaluated on DSC, XRD, and FT-IR. Morphological analysis and in vitro release study of the formulation was carried out. Next, the success of formulation toward anticancer potency against CC were performed via modulation NF-κB/mTOR/Bcl2/JNK/BAX/MDR1.

## 2. Experimental Methodology

### 2.1. Material

AGN, PLGA and D-α-tocopherol polyethylene glycol 1000 succinate (Vit E TPGS) were purchased from Sigma Aldrich, St. Louis, MO, USA. Lipoid SPC (hydrogenated phosphatidylcholine from soybean) was procured from LIPOID, (GmbH, Ludwigshafen Germany). Dichloromethane (DCM) and dimethyl sulfoxide (DMSO, 99.9%) were procured from Fischer Scientific (Loughborough, UK). A human colorectal cancer cell line was purchased from ATCC (Manassas, VA, USA). HCT 116 cells were grown in Dulbecco’s modified Eagle medium (DMEM, Gibco, London, UK), which was supplemented with 10% fetal bovine serum (FBS), Pen Strep (5000 units/mL penicillin and 5000 µg/mL streptomycin). The Annexin V/PI apoptosis detection kit was procured from Invitrogen Corporation (Carlsbad, CA, USA). 

### 2.2. Method of Preparation of LPHyNPs

LPHyNPs were prepared by nanoprecipitation method [22] with slight modification and in this case, solutions were designed in two-phase system. In phase one, 50 mg of PLGA (50:50), 100 mg of Lipoid S PC-3, and 5 mg of AGN (previously dissolved in 100 µL of DMSO) were dissolved in 5 mL of DCM. Whereas, in phase two, AGN/PLGA weight ratio was kept at 1:10 *w*/*w*, and the Lipoid: PLGA weight ratio was 2:1 *w*/*w*. Vit E-TPGS 1000 was dispersed in 10 mL of Milli-Q water at 0.5% *w*/*v* at heated to 70 °C. Then phase one solution was added drop-wise (at the rate of 1.5 mL/min) into the preheated phase two solution with magnetic stirring (500 rpm). The mixed solution was then homogenized (T25 digital Ultra-Turrax, IKA, UK) for 2 min (21,000 rpm), followed by magnetic stirring at 500 rpm for 4 h at 25 ± 1 °C for the complete evaporation of DCM. The final formulation was washed with Milli-Q water by ultracentrifugation at 30,000 rpm for 30 min (three cycles). Using the dialysis technique (Spectra/PorVR dialysis membrane), the prepared LPHyNPs were recovered and purified. The final LPHyNPs formulation (100 µL) was diluted 50-fold with Milli-Q water for particle characterization by dynamic light scattering (DLS) measurement, such as particle size, PDI, and zeta potential. Further, the formulations were lyophilized using mannitol (1%, *w*/*v*) as a cryoprotectant, and it was frozen at −80 °C and subjected to freeze-drying for further characterization.

### 2.3. Characterization of Prepared LPHyNPs

#### 2.3.1. Determination of Particle Size, PDI and Zeta Potential

Particle size, PDI, and zeta potential measurements were carried out via the DLS technique using the Zetasizer Nano ZSP instrument (Malvern Instruments Ltd., Malvern, UK). The particle size and PDI of three individual collected samples were analyzed after 50-fold dilution in Milli-Q water at a temperature of 25 ± 0.5 °C [23].

#### 2.3.2. Drug Entrapment and Loading Efficiency of Prepared LPHyNP

Drug entrapment (EE) and loading efficiency (DL) of prepared LPHyNP were analyzed in the supernatant (indirect method) of collected samples [24]. Approximately 5 mL of LPHyNPs was diluted in 5 mL of methanol to dissolve the drug and precipitate the PLGA and other excipients. The suspension was centrifuged (rpm for 20 min at 4 °C), and the supernatant was collected. To analyze the drug concentration in the collected supernatant, a 30 µL sample was injected into the HPLC-UV system. For this purpose, a chromatographic technique was developed, which contained a C_18_ analytical column (5 μm, 250 mm × 4.6 mm). The mobile phase was composed of acetonitrile and 0.1% formic acid at 55:45 (*v*/*v*), where pH was maintained at 7.4. The mobile phase was pumped isocratically at a 1 mL/min flow rate, and UV-detection was performed at 270 nm. The %EE and %DL were calculated as per the following equations:

%EE = [(Initial amount of drug − Amount of drug in supernatant)/Initial amount of drug] × 100

%DL = [(Initial amount of drug − Amount of drug in supernatant)/Initial amount of LPHyNPs] × 100

#### 2.3.3. Differential Scanning Calorimeter (DSC) Analysis

A differential scanning calorimeter (DC-60 plus; Shimadzu, Japan) was used to investigate the thermal characteristics of AGN, LPHyNP, trehalose, PLGA, SPC-3, and TPGS samples. In this case, the HyNPs were dried overnight in a desiccator, and then powdered samples were sealed in aluminum pans with lids and heated from a temperature of 25–300 °C with a rate of 10 °C/min under nitrogen flow [17].

#### 2.3.4. X-ray Diffraction Analysis

The samples of AGN, LPHyNPs, PLGA, SPC-3, trehalose, and Vit-E-TPGS were collected to determine their crystallinity. The degree of crystallization was analyzed using a high-resolution XRD (Maxima XRD-7000X, Shimadzu, Kyoto City, Japan) technology with an XRD scanning speed of 5–80°/min [25].

#### 2.3.5. FT-IR Analysis

For FT-IR analysis, various collected samples such as AGN, LPHyNPs, PLGA, SPC-3, and Vit-E-TPGS were analyzed using an FT-IR spectrophotometer (Bruker 375 Tensor-27, Billerica, MA, USA). Additionally, dry samples were compressed as KBr pellets using an instrument pin. The selected transmission range was between a wave number of 4000–400 cm^−1^ [26].

#### 2.3.6. Morphological Analysis

Scanning electron microscopy (SEM, ZEISS, Germany) and transmission electron microscopy (TEM, JEOL JEM 1010, Tokyo, Japan) were used to examine the nanostructure of the prepared LPHyNP. SEM is a high-resolution field emission scanning electron microscope that allows samples to be examined with an accelerating voltage of 15 kV [27]. Whereas, for TEM investigation, one drop of LPHyNP dispersion, which was prepared in Milli-Q water was dried on a copper grid. Uranyl acetate (2% *w*/*w*) was then used to stain the sample. The stained sample was air-dried to remove excess liquid media before being analyzed by TEM [28]. The length measurement of the particles was carried out with the help of ImageJ software (Version 1.53e, National institute of health, Bethesda, MD, USA). The particles are not exactly circular, so the length measurements were done at four different angles in such a manner that the shape is equally divided into two parts for each measurement. All measurements were transferred to the OriginPro software (Version 8.5.0, OriginLab Corporation, Northampton, MA, USA). The data were plotted as a histogram to get the bin worksheet. The bin worksheet generated a column plot using Bin centers vs. Bin counts. Finally, the plot was fitted using the nonlinear curve fit command of origin, and Gaussian fit was chosen to get the bell-shaped curve of Gaussian fit of counts.

### 2.4. Study of In Vitro Release Pattern

A dialysis bag with a molecular weight cut-off of 12,000 Da was used to perform in vitro drug release. In a nutshell, the LPHyNP and AGN suspensions were placed individually in the dialysis bag, knotted, and immersed in the release medium. The release media (50 mL) in the study was phosphate-buffered saline (PBS) of pH 7.8 with sodium lauryl sulfate (1%) as a solubilizer. Throughout the investigation, the system was kept at 37 °C in a shaker water bath. The samples were taken at 1, 2, 3, 4, 6, 8, 24, 48, and 72 h, and the AGN content was analyzed at given time intervals using the HPLC technique [29].

### 2.5. Cell Viability Assay

For the assessment of the cell viability assay, a colon cancer cell line (HCT-116) was used, and MTT proliferation kit (Sigma Aldrich, St Louis, MO, USA) was used. Initially, the cells were incubated at the temperature of 37 °C using a 96-well plate for 24 h where cells were grown and media was supplemented with 10% FBS, 1% Pen/strep, and 1 mM glutamine. The density of cells used in the process was 5 × 10^3^ HCT-116 cells/well in a humidified CO_2_ chamber. Cells were treated with the concentration of 6.25, 25, and 100 µg/mL of blank NPs, AGN suspension, and LPNHyNP and left for 24 h and then treated with MTT solution. Stock solution was prepared by dissolving 5 mg of MTT in 1 mL or 1000 µL of PBS. About 10 µL of the stock solution was used per well and kept for 4 h at 37 °C to develop formazan crystals. In the next step, excess culture media from each well was removed by washing and formazan crystals were solubilized by adding 100 μL DMSO for 20 min and the absorbance was recorded at 563 nm [30]. The same procedure mentioned above was used for a normal human cell line, i.e., HEK293 (the most widely used and readily available normal human cell line), to determine the cytotoxic effect of blank LPHyNPs, AGN suspension, and LPHyNPs formulations on a normal cell line. The formulations were treated in the same dose range as that used for the HCT-116 cell line.

### 2.6. Cell Cycle and Apoptosis Analysis Using Flow Cytometry

For the analysis of cell cycle, HCT-116 cells were properly fixed in 70% ethanol on ice for approximately 15 min. In the next step, HCT-cells were incubated in the binding buffer and propidium iodide at room temperature in a dark room for 20 min. Apoptosis assay was performed by using annexin V (FITC)/PI assay kit (K101-100, Biovision Inc., Milpitas, CA, USA) and performed as per the manufacturer’s instruction. In brief, 1 × 10^5^ cells/mL were allowed to get treated with HCT-116 and incubated for 24 h. Next, the cells were centrifuged, washed with the phosphate buffer, and resuspended using 500 µL of the buffer. Followed by this, 100 µL of resuspended cells were again incubated with the 5 µL of PI and Annexin-V at room temperature in a dark room for 15 min and analyzed using BD FACSCalibur reader, and data were analyzed using flow cytometer and flow system software, USA [31,32].

### 2.7. Gene Expression of the Various Carcinogenic Marker Using RT-PCR

For the estimation of BAX, Bcl-2, NF-κB, mTOR, JNK, and MD1, HCT-116 cells were initially treated with the various treatment groups at the concentration of IC50 of various samples and incubated in 96-well plated; blank NPs (124.77 µg/mL), AGN suspension (50.93 µg/mL), and LPNHyNPs (10.89 µg/mL). Concentration of IC50 was set in the present study because at this concentration maximum cytotoxicity was observed. In the RT-PCR study, to extract RNA, TRIzol reagent was used, and cDNA was synthesized. The expression level of the aforementioned genes was estimated using Rotor-Gene Q software and reported as fold change [30]. The primer sequence used in the study is shown in Table 1.

### 2.8. Statistical Analysis

The experiments were carried out in triplicates, and the findings were given as mean ± standard deviation (SD). A one-way ANOVA followed by Tukey, multiple comparison tests was used to determine statistical significance, with a *p*-value of <0.05 considered significant.

## 3. Results and Discussion

### 3.1. Determination of Particle Size, PDI, and Surface Potential of LPHyNP

**Figure 1:**
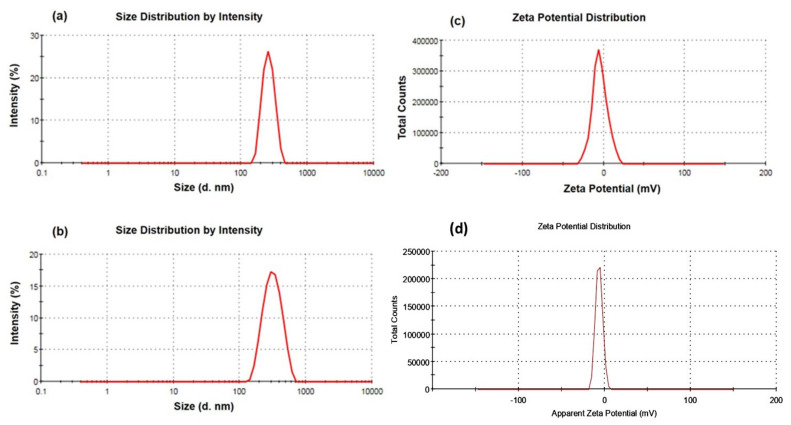
(**a**,**b**) Demonstrate particle size of blank LPHyNPs and drug-loaded LPHyNPs, and (**c**,**d**) show the zeta potential of blank LPHyNPs and drug-loaded LPHyNPs, respectively.

The DLS technique was used to determine the particle size, PDI, and zeta potential. The prepared blank LPHyNPs and drug-loaded LPHyNPs exhibited an average particle size of 200.26 ± 9.19 nm (Table 2) and 234.80 ± 12.28 nm (Figure 1a,b), respectively, which has been pondered as HyNPs. Simultaneously, the observed PDI of blank LPHyNPs and drug-loaded LPHyNPs were 0.34 ± 0.10 and 0.11 ± 0.04, respectively. These results were associated only with the addition of drug. When the drug was added, the particle size of NP was increased due to the entrapment of the drug, which expanded its particle size. On the contrary, the PDI of drug-loaded LPHyNPs decreased compared to blank LPHyNPs, which demonstrated a homogenous population of NP [33]. The average zeta potential of blank NPs and LPHyNPs were found to be −5.15 ± 0.70 mV and −4.14 ± 0.81, respectively (Figure 1c,d). Thus, the developed LPHyNPs was considered as a stable formulation. 

### 3.2. Drug Entrapment and Loading Efficiency of Prepared LPHyNP

The EE and DL of LPHyNPs were calculated to determine the drug concentration in the NP. The EE of LPHyNPs was recorded as 55.18 ± 3.61% (Table 2). In this case, the drug was generally entrapped inside the NP, which sustained the release of the drug from the LPHyNPs. Simultaneously, DL of LPHyNPs was recorded as 11.04 ± 0.72%, and this DL was due to AGN affinity toward the used polymer, i.e., PLGA.

### 3.3. Differential Scanning Calorimeter (DSC) Analysis

**Figure 2:**
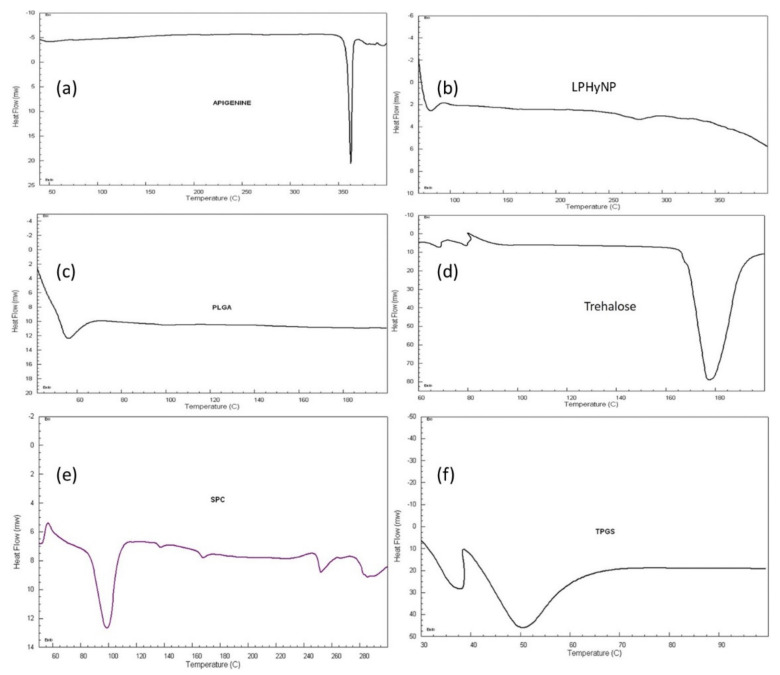
DSC thermogram of (**a**) AGN, (**b**) LPHyNP, (**c**) PLGA, (**d**) trehalose, (**e**) SPC-3, and (**f**) TPGS.

In this study, Figure 2 shows the DSC thermograms of AGN, LPHyNP, trehalose, PLGA, SPC-3, and TPGS. The thermogram of AGN showed a sharp endothermic peak at 320 °C, which demonstrated the crystallinity of drug molecules. Because of its glass transition temperature (Tg), the polymer shows an endothermic peak at 51.72 °C, whereas SPC-3 shows a gel–liquid crystalline phase transition at 43.28 °C, followed by a series of irregular peaks above 220 °C, indicating deterioration at higher temperatures [34]. The thermogram of drug-loaded LPHyNP demonstrated the distinctive peak of PLGA and TPGS at 40 °C, the short peak of trehalose at 270 °C, and the absence of AGN peak confirmed the encapsulation of the drug inside the NP.

### 3.4. X-ray Diffraction Analysis

**Figure 3:**
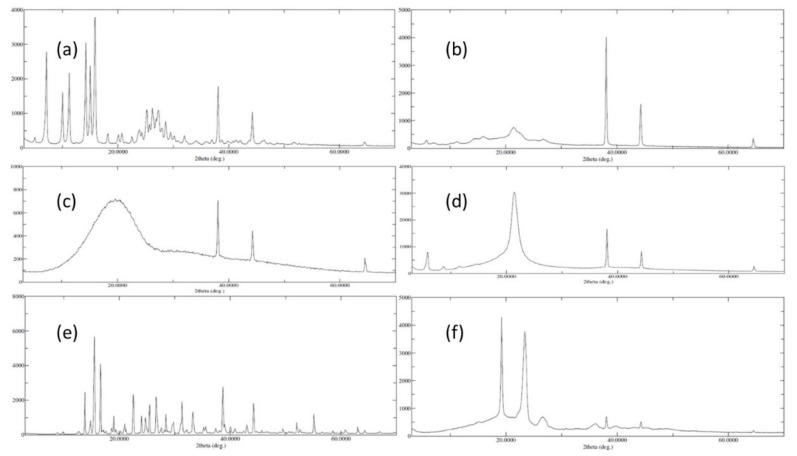
XRD diffractograms of (**a**) AGN, (**b**) LPHyNP, (**c**) PLGA, (**d**) SPC-3, (**e**) trehalose, and (**f**) TPGS.

An XRD examination was carried out to determine the crystallinity of different collected samples, such as AGN, LPHyNP, PLGA, SPC-3, trehalose, and Vit-E-TPGS. Figure 3 shows the XRD diffractogram of these samples, in which Figure 3a revealed well-defined peaks of AGN in the examined range, which could reflect the AGN powder’s crystalline composition. The lack of unique diffraction peaks in PLGA (Figure 3c) and SPC-3 (Figure 3d) can be attributed to their amorphous natures. On the other hand, the trehalose showed some of the well-defined peaks of the crystalline nature of the material (Figure 3e). Figure 3f shows some of the TPGS and Vit-E characteristic diffraction peaks in their physical mixing. Additionally, Figure 3b claims some of the AGN characteristic diffraction peaks with noisy PLGA and SPC-3 peaks. The amorphous form of the particles was confirmed by the XRD of the LPHyNP.

### 3.5. FT-IR Analysis

**Figure 4:**
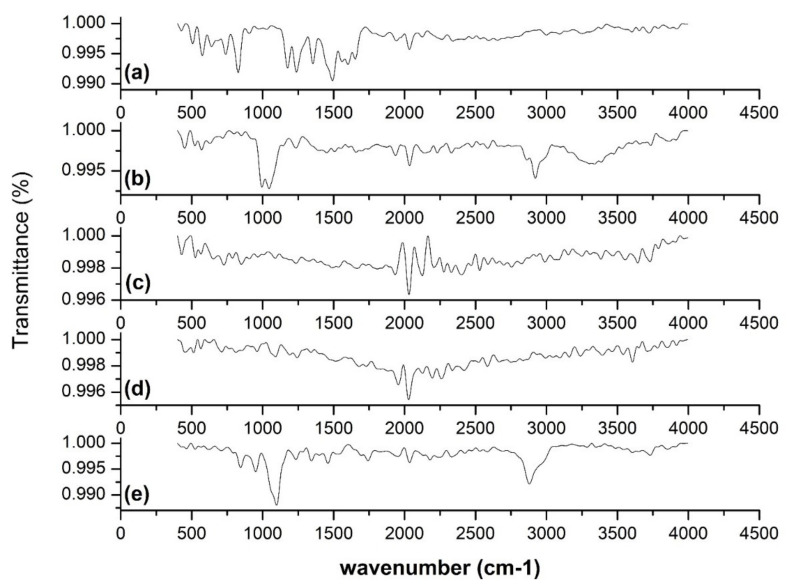
FT-IR spectra of (**a**) AGN, (**b**) LPHyNP, (**c**) PLGA, (**d**) SPC-3, and (**e**) TPGS.

To determine the comparative difference between free AGN and developed LPHyNPs, an FT-IR spectroscopy study was carried out, and spectra of various tested samples (AGN, LPHyNPs, PLGA, SPC-3, and Vit-E-TPGS) are shown in Figure 4. FT-IR spectra show the existence of specific functional groups in the AGN (Figure 4a). The characteristic bands of AGN were found at 3386.44, 1652.39, 1491.80, 1237.89, 1026.76, and 972.59 cm^−1^. The absorption at 3386.44 cm^−1^ and 1652.39 cm^−1^ presented stretching vibration of the hydroxyl group (OH) and C=O stretching toward the lower vibrational frequencies. The absorption at 1491.80 cm^−1^ was attributed to CH_2_ stretching vibration. The absorption at 1237.89 cm^−1^ (antisymmetric stretching) and 1026.76 cm^−1^ (C-O-P-O-C stretching) indicated polar head group vibration. The absorption peak at 971 cm^−1^ exhibited the antisymmetric N^+^-CH_3_ stretching vibrations [35]. The FT-IR spectra of developed LPHyNPs (Figure 4b) demonstrated a broad absorption peak at 3385.32 cm^−1^ due to the stretching vibrations of the OH group of PLGA and SPC-3. The stretching peak at 2920.81 cm^−1^ presented the stretching vibration of C-H. A peak at 1044.37 cm^−1^ was also observed, which showed the C-O stretching of TPGS. Hence, the FT-IR spectra of LPHyNP indicated promising drug entrapment in the polymer.

### 3.6. Morphological Analysis

**Figure 5:**
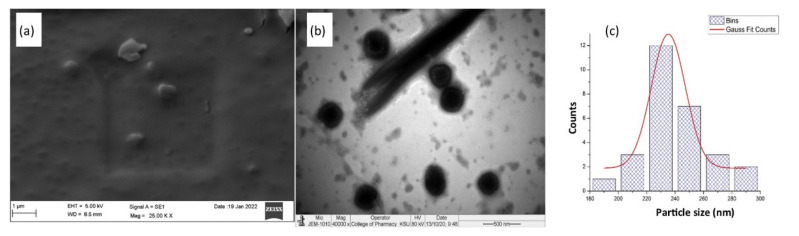
Morphological studies of LPHyNPs (**a**) SEM image, (**b**) TEM image, (**c**) size histogram of TEM.

The morphological pattern of developed LPHyNP obtained via SEM study is depicted in Figure 5a. In the SEM image, LPHyNP exhibited a solid and asymmetrically shaped particle with a smooth surface. Concurrently, the TEM image (Figure 5b) also demonstrated asymmetrically shaped particles with more or less uniform size. The light gray spots in the TEM image indicated unreacted components of the formulation. Additionally, the size histogram (Figure 5c) of TEM showed the average particle size of optimized LPHyNP between 235 and 240 nm.

### 3.7. Study of In Vitro Release Pattern

**Figure 6:**
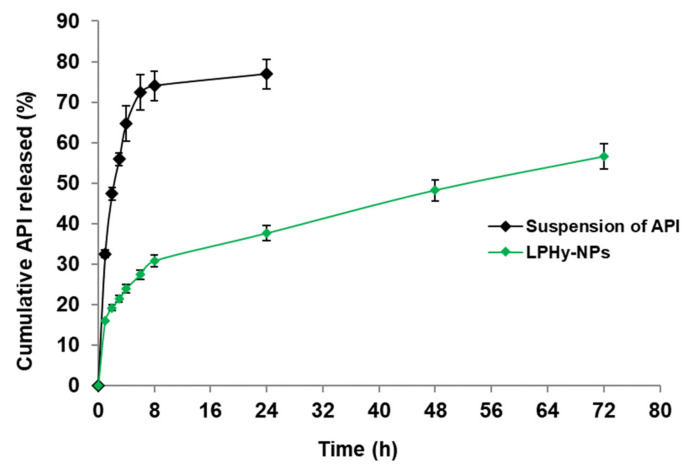
In vitro release of AGN from LPHyNP and suspension of AGN in PBS containing SLS (1%) as a solubilizer by dialysis bag method.

In vitro drug release study was performed using the dialysis bag method to analyze the release pattern of LPHyNPs and suspension of AGN, and the result is shown in Figure 6. The release of AGN from LPHyNPs was sufficient for 72 h. In contrast, AGN was released entirely within 24 h with a fast release pattern from the suspension of AGN. In the case of AGN release from LPHyNPs, an initial burst release was observed for up to 8 h followed by sustained release till 72 h. Therefore, LPHyNPs released AGN significantly (*p* < 0.05) better than the suspension of AGN. Thus, the PLGA encapsulation of LPHyNPs controls the release and provides a sustained release pattern for more than 64 h. The encapsulation of lipophilic AGN in the core of HyNP-contained PLGA could be ascribed to such a sustained release behavior [36].

### 3.8. Cell Viability Analysis (MTT Assay)

**Figure 7:**
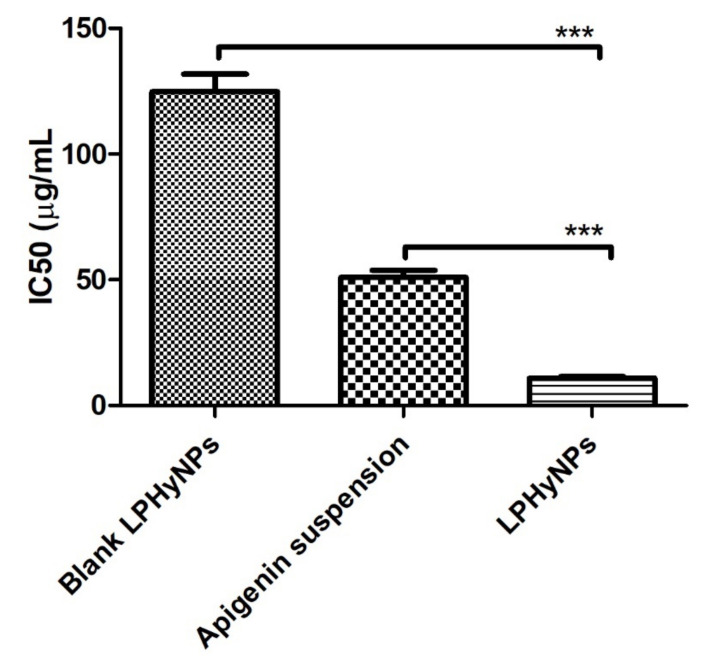
Showing the outcome of MTT assay for blank LPHyNPs, AGN suspension, and LPHyNPs. Values are presented as mean ± SD where *p* < 0.001) was represented by ***.

MTT, or cell viability, is one of the extensively studied parameters during the initial phase of drug development in preclinical studies. MTT assay provides reliable and concrete information for the cell viability and proliferation property of the drug. In this process, when the drug is exposed to the MTT solution, the viable cells undergo fragmentation of DNA, get colorized, and absorbance is recorded. Upon quantifying the recorded absorbance, viability, as well as IC_50_ concentration in µg/mL, was calculated [37]. The lower the IC_50_ concentration, the more potential is the drug. Here, biosafety and cytotoxicity of blank LPHyNPs, AGN suspension, and LPHyNPs were evaluated on HEK293 and HCT-116. The prepared samples were tested in the same dose range as that tested for the colon cancer cell line, and the cytotoxicity was recorded as minimal and non-significant (*p* < 0.05), as shown in Supplementary Figure S1. Simultaneously, cell viability study was also performed on selected colon cancer cell line (HCT-116). In the current study, when HCT-116 cells were treated with the various drug formulation, and it was found that IC_50_ of blank LPHyNPs was 124.77 ± 7.01 µg/mL, AGN suspension showed IC_50_ of 50.93 ± 2.86 µg/mL, and LPHyNPs exhibited IC_50_ 10.89 ± 0.61 µg/mL, respectively (Figure 7). Thus, as discussed above, the observed results showed that LPNHyNPs was 5 times more potent than AGN suspension and 12 times more potent than blank LPHyNPs. So, it was concluded that the significantly higher anticancer potential of LPNHyNPs could be due to fabricated nanoformulation that exhibits superior drug penetration into the tumor cells. Moreover, the blank nanoformulation also showed anticancer activity due to the presence of Vit E-TPGS 1000 [38].

### 3.9. Effect of LPHyNPs on Cell Cycle

The uncontrolled division is one of the hallmarks of the initiation and progression of tumorigenesis. Although, in a normal homeostatic condition, the frequency of cell division is almost balanced by the apoptotic and necrotic events as an optimum cellular number and volume are critical for the smooth working of various vital organs. Multiple signaling pathways and molecules such as retinoblastoma protein (pRb), cyclin-dependent kinases (Cdk), CDK-activating kinase complex (CAK), etc., are needed for the continuous and balanced cell cycle [39]. However, in response to various oncogenic stimulus, disbalance occurs among these molecules, and an uncontrolled cell cycle occurs. Thus, more detailed and exploratory studies were performed to understand the multiple checkpoints, and likewise, a number of clinically used drugs were developed. At present, various FDA-approved drugs have been used that target the various cell cycle phases [40]. Thus, in the present study, we have also performed the cell cycle analysis to establish the anticancer effect of the developed formulation against colon cancer. Based on the outcome of cell cycle analysis, it was found that a maximum percentage of the cell of the control was present in the G0–G1 phase and exhibited proliferator activity. Moreover, no significant difference was found for the percent cell in the G0–G1 phase when treated with blank LPHyNPs, AGN suspension, and LPHyNPs. 

In the present study, the cell population of LPHyNPs in the G2 phase is extremely higher than in the control and the AGN. As it is well-known that the accumulation of cells in the G2/M phases is a distinguishing feature of apoptosis and cell cycle arrest at the G2 checkpoint. Hence, the significant pro-apoptotic effect of LPHyNPs was confirmed and the result of the apoptosis assay was also validated. Hence, the finding of the current study indicates that the effect of LPHyNPs on activating apoptosis is related to an increased proportion of cells at the G2/M phases. Similar findings were observed by others. In other words, an increase in the cell population at the G2-M phase signifies that the relegation event is inhibited that follows DNA cleavage by topoisomerase II. This effect triggers the accumulation of double-strand breaks (loss of heterozygosity and chromosome rearrangements that result in the cell) in the genome after it is replicated, thus producing cell cycle arrest at the G2-M phase. Hence, increase in cell population by LPHyNPs in the G2-M phase signifies the potential of LPHyNPs to cause cell cycle arrest.

**Figure 8:**
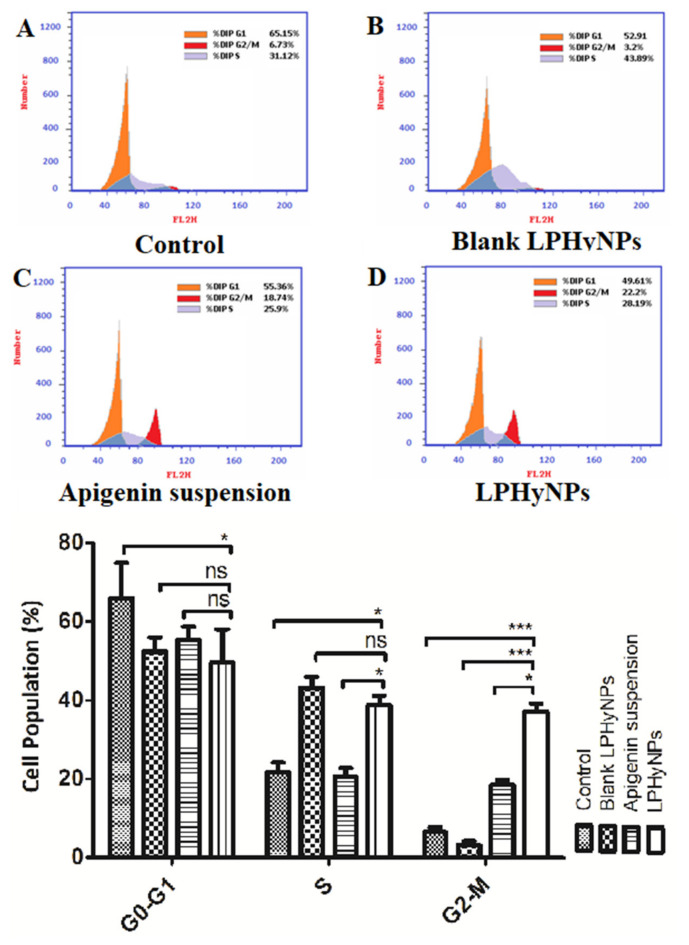
Figure 8 showing the outcome of cell cycle analysis using flow cytometry where effect of different groups such as (**A**) control, (**B**) Blank LPHyNPs, (**C**) apigenin and (**D**) LPHyNPs were evaluated. Values are presented as mean ± SD where *p* < 0.05 and *p* < 0.001 was represented by * and ***. ‘ns’ demonstrated non-significance differences between results.

Furthermore, it is well established that the S phase is most crucial for the smooth continuation of the cell cycle, and in this phase only, the amount of DNA doubles. Thus, blockage of this phase is ideal for a potent anticancer drug. A maximum percent of cells were found in the S and G2-M phases. A significant difference was found between AGN suspension (*p* < 0.05) and LPHyNPs, as shown in Figure 8. Thus, the superiority of LPHyNPs was established in terms of increased apoptotic and necrotic activity. In a nutshell, LPHyNPs and AGN suspension arrest cell growth at G2/M phase, whereas blank NPs arrest cell growth at S phase and hence, the rationale for formulating LPHyNPs was justified.

### 3.10. Apoptosis Assay Using Flow Cytometry

**Figure 9:**
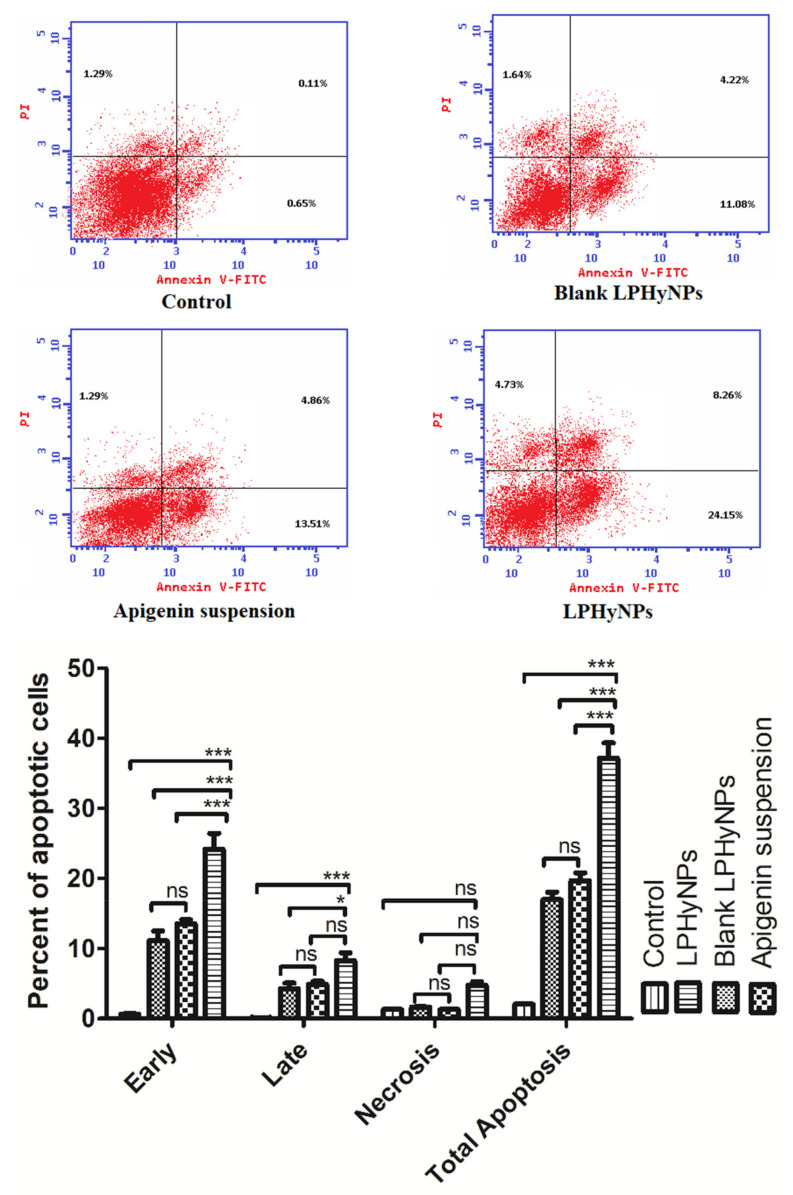
Showing the pro-apoptotic effect of various drug formulations such as control, Blank LPHyNPs, apigenin and LPHyNPs against colon cancer cell lines using flow cytometry. Values are presented as mean ± SD where *p* < 0.05 and *p* < 0.001 was represented by * and ***. ‘ns’ demonstrated non-significance differences between results.

Apoptosis is also known as programmed cell death, and it is one of the decisive parameters used to estimate the anticancer potential of a drug entity. Undoubtedly, apoptosis plays a major role in maintaining normal physiological function via maintaining the number and volume of cells [23]. However, when the normal pace of apoptosis is disturbed in response of carcinogenic stimulus, programmed cell death is abrogated, and uncontrolled cell division and survival of tumor cells take place, leading to the initiation and progression of the carcinogenic event. In line with this, numerous published pieces of evidence have shown a positive correlation between reduced apoptosis and progression of colon cancer. Thus, estimation of an apoptotic event or apoptotic cells provided a substantial idea about the progression of disease etiology and also helped in assessing the clinical out of anticancer drugs [41]. Moreover, the apoptotic assay is also regularly performed to validate the MTT assay outcome. The clinical relevance of apoptotic assay can be understood from the fact that many apoptosis-stimulating drugs are in different phases of the clinical trial, and many of the existing anticancer drugs act via stimulating apoptosis in the cancerous cells. Thus, looking into the clinical relevance of apoptosis, in the present study, we have also studied the apoptotic activity of various drug formulations using flow cytometry. Upon comparing the necrotic effect, it was found that control, blank LPHyNPs, and AGN suspension showed almost comparable effects. In contrast, LPHyNPs exhibited significantly superior necrotic effect (1.29%, 1.69%, 1.29% vs. 4.73%) as shown in Figure 9. When the late apoptotic activity was studied, it was found that LPHyNPs showed almost ~2 times higher apoptotic activity as compared to blank LPHyNPs (Q2: 4.22%, blank LPHyNPs) and AGN suspension (4.86%, AGN suspension) and 8.26% (LPHyNPs). Similarly, when the early apoptotic activity was estimated, we found that LPHyNPs showed almost ~2 times higher apoptotic activity as compared to blank LPHyNPs and AGN suspension that, on the other hand, showed similar apoptotic activity (Q4: 11.08%, blank LPHyNPs; 13.51%, AGN suspension) and 24.15% (LPHyNPs). Moreover, upon comparing the total apoptotic activity, it was found that AGN suspension exhibited 1.16 times higher apoptotic activity as compared to blank LPHyNPs, whereas LPHyNPs showed 2.19 and 1.89 times higher total apoptotic activity as compared to blank LPHyNPs and AGN suspension (Q4: 16.94%, blank LPHyNPs); 19.66% (AGN suspension) and 37.14% (LPHyNPs) as shown in Figure 9.

Based on the apoptotic activity it was found that blank NPs showed higher apoptotic activity as compared to control and blank NPs’s apoptosis rate is due to its component “Vit E-TPGS 1000” that has proven anticancer property. Vit E-TPGS 1000 was added in blank NPs because of its solubilizer, emulsifier, stabilizer properties, absorption, permeation enhancer and its property to inhibit P-gp overexpression. Thus, in conclusion necrotic activity, early, late, and total apoptotic activity was significantly higher for LPHyNPs as compared AGN suspension. Hence, the LPHyNPs was found to be potential and effective in ameliorating the colonic cancerous cells by stimulating apoptotic activity. 

### 3.11. Effect of LPHyNPs on the Gene Expression of Bcl-2, BAX, NF-κB, mTOR, JNK, and MDR-1

**Figure 10:**
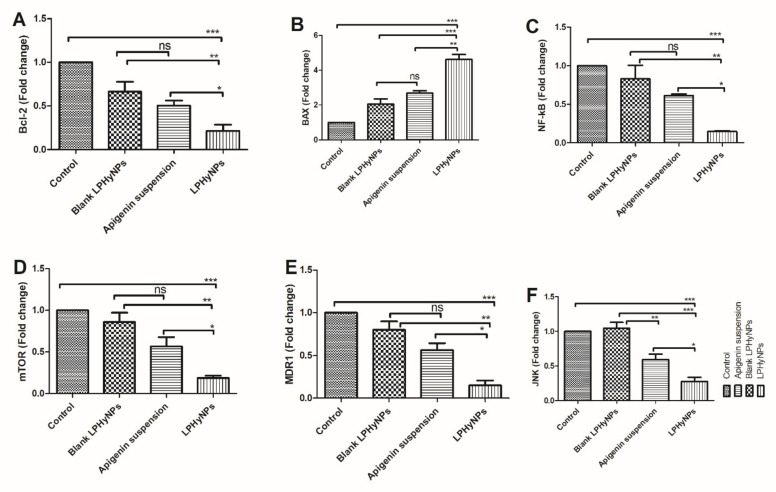
Showing the the outcome of RT-PCR on various formulations such as blank LPHyNPs, AGN suspension, and LPHyNPs. (**A**) represent mRNA expression of Bcl-2, (**B**) represent mRNA expression of BAX, (**C**) represent mRNA expression of NF-kB, (**D**) represent mRNA expression of mTOR, (**E**) represent mTNs expression of MDR1 and (**F**) represent mRNA expression of JNK. Values are presented as mean ± SD where * (*p* < 0.05: *p* < 0.01 and *p* < 0.01) was represented by *, **, and ***. ‘ns’ demonstrated non-significance differences between results.

At present, the importance of apoptosis and modulation of various pro and anti-apoptotic genes are well established in the etiology of colon cancer. In a normal physiological condition, a balance is maintained between pro and anti-apoptotic genes. In contrast, during the cancerous phase, apoptotic activity is significantly reduced along with the reduction in expression of pro-apoptotic genes and elevation in the expression of anti-apoptotic genes. In the present study, we have already shown that overall apoptotic activity was reduced, and treatment in the LPHyNPs significantly elevates the apoptotic activity. Hence, we have tried to further investigate the mechanism and expression of various genes modulated upon exposure to different treatment groups. In general, apoptosis is regulated by proteins, namely caspase-3, caspase-6, cytochrome c, BAX, and Bcl-2 [42]. Numerous published evidences have shown increased expression level of Bcl-2 (anti-apoptotic gene) and reduced expression of BAX (pro-apoptotic gene), whereas, upon treatment with a potential anticancer drug, their level gets reversed, and anticancer effect is achieved. Bcl-2, an anti-apoptotic protein, mitigates apoptotic mechanism and supports the cancerous cell in undergoing uncontrolled cell division and survival. BAX, on the other hand, being a pro-apoptotic protein, gets attached to the anti-apoptotic proteins, sequesters their anti-apoptotic activity, stimulates the release of cytochrome C, and eventually promotes/stimulates apoptosis leading to the exhibition of anticancer effect [43]. In the present study, when blank LPHyNPs, AGN suspension, and LPHyNPs were treated with HCT-116 cells, LPHyNPs showed a significant reduction in the expression level of Bcl-2 and elevation in the expression of BAX when compared with AGN suspension (*p* < 0.05 for Bcl-2 and 0.01 for BAX) and blank LPHyNPs (*p* < 0.01 for Bcl-2 and *p* < 0.001 for BAX). Henceforth, the outcome of this study validated the rationale for formulating LPHyNPs as shown in Figure 10.

Apart from the direct involvement of the apoptotic mechanism in the pathogenesis of colon cancer, the profound role of the inflammatory pathway has also been well established. Among various contributors of inflammation during carcinogenesis, NF-κB is one of the extensively studied transcription factors. During the normal physiological function, NF-κB remains sequestered in the cytoplasm associated with the ikB. Upon exposure to the carcinogenic stimulus, the inhibitory effect of ikB is withdrawn due to phosphorylation. NF-κB undergoes nuclear translocation and regulates the expression of inflammatory signaling molecules such as TNF-α, IL-6, IL-1β, COX-2, etc. Additionally, increased expression of NF-κB has been positively correlated with the enhanced angiogenesis, tumor invasion, and metastasis, where it modulates MMPs and VEGF activity [44]. Thus, a potent drug candidate ideally reduces NF-κB expression and exhibits a potent anticancer effect. We also analyzed the expression level of NF-κB upon treatment with the various formulation. The outcome of the study showed a significant reduction in the expression level of NF-κB when treated with LPHyNPs as compared to blank LPHyNPs (*p* < 0.05) and AGN suspension (*p* < 0.01), as shown in Figure 10.

Apart from NF-κB, mTOR is yet another extensively studied signaling molecule reported to have a potential pathogenic role in colon cancer. Undoubtedly, mTOR in a normal physiological process plays numerous biological functions via its cross-talk with PI3K/Akt/MAPK/NF-κB and p53, showing its involvement in the colon and other types of cancer via regulation of tumor cell cycle, survival, proliferation, invasion, and metastasis. Studies have shown that the dysregulated mTOR pathway is related to a gene mutation, leading to its increased expression and continuous hyperactivation. Moreover, the pathogenic role of the mTOR pathway was further confirmed in the studies where the use of its inhibitor exhibited a potent anticancer effect. The combination of mTOR inhibitor and conventional drugs is currently being investigated to achieve a superior clinical outcome in different types of cancer [45]. 

No doubt, the role of potent pharmacotherapy is needed at this time for the management and treatment of colon cancer. But at the same time, drug resistance is yet another hindrance in achieving the optimum clinical outcome. Many potent anticancer drugs fail to achieve desired clinical response just because of drug resistance. Recently, MDR-related genes such as MDR1 and JNK have gained the attention of researchers because of their role in promoting carcinogenic events [46]. c-Jun N-terminal kinase (JNK) is an important signaling molecule reported to be involved in the pathogenesis of colon and other types of cancer. Published evidence has reported the pro-carcinogenic role of these molecules. It acts in close proximity with NF-κB/mTOR/JAK-STAT signaling pathway and inhibits apoptosis, regulates autophagy, and eventually promotes tumor cell survival [47]. A more detailed study showed that JNK also plays a critical role in tumor evasion, modulating the expression of p21, p53 c-Myc, and drug resistance. MDR-1 is one of the important genes involved in the multidrug resistance of various anticancer drugs, and the exploratory studies have shown significant elevation in its expression in various types of cancer. Increased expression of MDR-1 has also been positively correlated with elevated Bcl-2 and JNK expression [48]. Hence, in the present study, we explored the anticancer effect of prepared LPHyNPs via the modulation of expression of mTOR, JNK, and MDR-1 genes. The outcome of the study showed a significant reduction in the expression level of mTOR, JNK, and MDR-1 when treated with LPHyNPs as compared to blank LPHyNPs (*p* < 0.05 for mTOR, MDR-1, and JNK) and AGN suspension (for mTOR, MDR-1, and JNK) as shown in Figure 10.

## 4. Conclusions

The outcome of the present study showed the successful preparation of LPHyNPs. In the current study, LPHyNPs was prepared and characterized on various parameters particle size, PDI, zeta potential, and EE. Further, prepared LPHyNPs was evaluated on DSC, XRD, and FT-IR, and the outcomes of the studies confirmed the entrapment and structure of NP. The in vitro release study provided data of the sustained release of AGN from LPHyNPs. The success and rationale of developing LPHyNPs against colon cancer were studied and validated via performing flow cytometry to estimate apoptotic activity, where a significant and superior anticancer effect was achieved compared to blank LPHyNPs and AGN suspension. The mechanism behind the anticancer attribute was further explored via estimation of gene expression of various signaling molecules such as Bcl-2, BAX, NF-κB, and mTOR that are involved in carcinogenic pathways. Moreover, to strengthen the anticancer potential of LPHyNPs against chemoresistance, the expression of JNK and MDR-1 genes were estimated. It was found that their expression level reduced considerably when compared to blank LPHyNPs and AGN suspension. However, more detailed investigational cellular and molecular studies are needed to provide substantial evidence to bring this developed formulation from bench to bedside.

## Figures and Tables

**Table 1:** The primer used in the RT-PCR.

Target	Primer Used
**BAX**	F: 5′-CTGCAGAGGATGATTGCCG-3′ R: 5′-TGCCACTCGGAAAAAGACCT-3′
**Bcl-2**	F: 5′-GACTTCGCCGAGATGTCCAG-3′ R: 5′-GAACTCAAAGAAGGCCACAATC-3′
**mTOR**	F: 5′-GCTTGATTTGGTTCCCAGGACAGT3 R: 5′-GTGCTGAGTTTGCTGTACCCATGT3′
**JNK**	F: 5′ -GTGT-GGAATCAAGCACCTTC-3′ R: 5′ -AGGCGTCATCATAAAACTCGTTC-3
**NF-κB**	F: 5’- CGCATCCAGACCAACAACA-3’ R: 5’- TGCCAGAGTTTCGGTTCAC-3’
**MDR1**	F: 5′-CCC ATC ATT GCA ATA GCA GG-3′ R: 5′-TGT TCA AAC TTC TGC TCC TGA-3′
**β-actin**	F: 5′-AGAGCTACGAGCTGCCTGAC-3′ R: 5′-AGCACTGTGTTGGCGTACAG-3′

**Table 2:** Result of characterization of blank LPHyNPs and drug-contained LPHyNPs.

Formulation	Particle Size (nm)	PDI	Zeta Potential (mV)	EE (%)	DL (%)
**Blank LPHyNPs**	200.26 ± 9.19	0.34 ± 0.10	−4.14 ± 0.81	-	-
**LPHyNPs**	234.80 ± 12.28	0.11 ± 0.04	−5.15 ± 0.70	55.18 ± 3.61	11.04 ± 0.72

## Data Availability

The data presented in this study are available in article.

## Supplementary Materials

The following supporting information can be downloaded at: https://www.mdpi.com/article/10.3390/polym14173577/s1, Figure S1: Cytocompatibility study of blank LPHyNPs, AGN suspension, and LPHyNPs formulations on HEK293 normal cell line via MTT assay. Values are presented as mean ± SD (n = 3).
