# Supplementary material for: Apigenin Loaded Lipoid–PLGA–TPGS Nanoparticles for Colon Cancer Therapy: Characterization, Sustained Release, Cytotoxicity, and Apoptosis Pathways
Source: Polymers (Basel). 2022 Aug 30;14(17):3577. doi: 10.3390/polym14173577 (PMC9460590; doi:10.3390/polym14173577)
Supplement: Supplementary file 1 [file polymers-14-03577-s001.zip › polymers-1787388-supplementary.pdf]

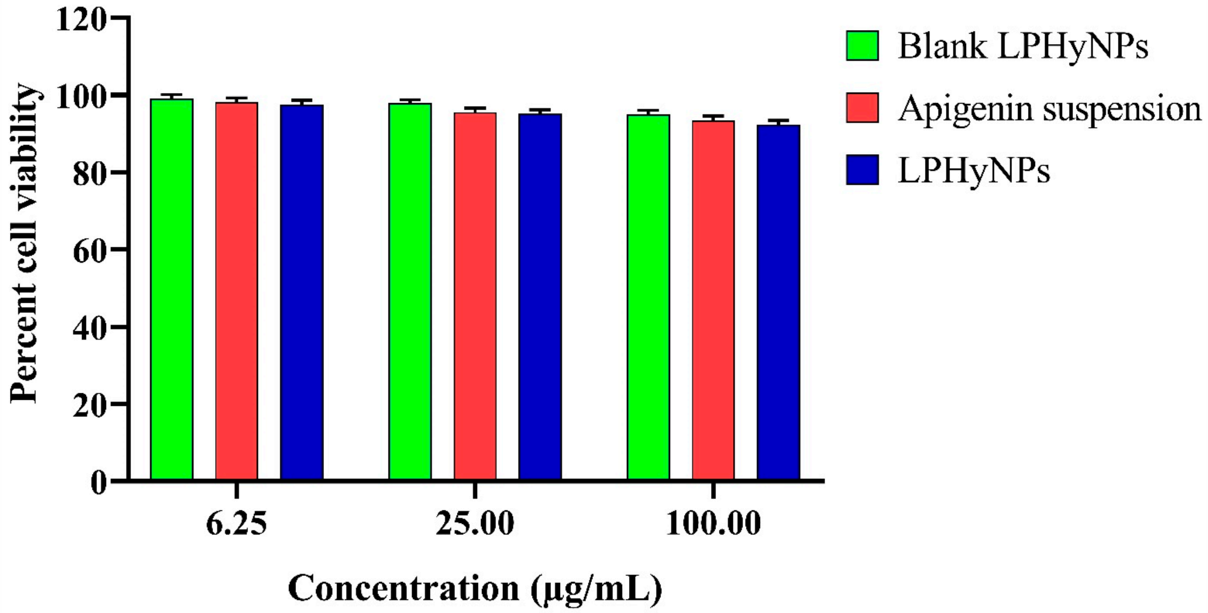

Supplementary Figure S1. Cytocompatibility study of blank LPHyNPs, AGN suspension, and LPHyNPs formulations on HEK293 normal cell line via MTT assay. Values are presented as mean ± SD (n=3).
